# Clinical Data Analytics With Time-Related Graphical User Interfaces: Application to Pharmacovigilance

**DOI:** 10.3389/fphar.2018.00717

**Published:** 2018-08-30

**Authors:** Thibault Ledieu, Guillaume Bouzillé, Elisabeth Polard, Catherine Plaisant, Frantz Thiessard, Marc Cuggia

**Affiliations:** ^1^INSERM, UMR 1099, Rennes, France; ^2^Université de Rennes 1, LTSI, Rennes, France; ^3^CHU Rennes, Rennes, France; ^4^Human-Computer Interaction Lab, University of Maryland, College Park, College Park, MD, United States; ^5^INSERM, Bordeaux Population Health Research Center, Team ERIAS, UMR 1219, Université de Bordeaux, Bordeaux, France

**Keywords:** informatics, graphical user interface, temporal data mining, pharmacovigilance, usability testing

## Abstract

Pharmacovigilance consists in monitoring and preventing the occurrence of adverse drug reactions. This activity can be time-consuming because it requires the collection of both patient and medication information. In this paper, we present two visualization and data mining applications to make this task easier for the practitioner. These tools have been developed and tested using the biomedical data warehouse eHOP (Hospital Biomedical Data Warehouse) of the Rennes University Hospital Centre. The first application is a tool to visualize the patient electronic health record in the form of a timeline. All patient data is collected and displayed chronologically. The usability test of the timeline has been very positive (SUS score: 82.5) and the tool is now available for practitioners in their daily practice. The second application is a tool to visualize and search the sequences of a patient cohort. The visual interface allow user to quickly visualize sequences. A query builder allows user to search for sequences in relation with a reference sequence, such as a prescription sequence followed by an abnormal biological value. The sequences are then visually aligned with this reference sequence and ranked by similarity. The GSP (Generalized Sequential Pattern) and Apriori algorithms allow us to display a summary of the sequences list by searching for common sequences and associations. The tool was tested on a use case which consisted in detection of inappropriate drug administration. Compared to a random order, we showed this ranking system saved the practitioner time in this task (to analyze one sequence, 3.49 ± 3.54 vs. 2.26 ± 2.86 s, *p* = 0.0003). These two visualization and data mining applications will help the daily practice of pharmacovigilance.

## Introduction

### Pharmacovigilance Context

The emergence of clinical data warehouses (CDW) has facilitated the re-use of patient data. These systems allow exploiting the variety and volume of clinical data. eHOP (Cuggia et al., 2011) and i2b2 (Murphy et al., 2010) are among the best known CDW examples. Clinical data are heterogeneous in terms of structure (structured or textual data) and domain (e.g., clinical/laboratory results or prescription data). Searching and organizing such information require specific expertise and is often a time-consuming task due to the lack of adapted tools. Data visualization can help to analyze and develop hypotheses based on massive and complex data.

Pharmacovigilance is defined by the World Health Organization as “the science and activities relating to the detection, assessment, understanding and prevention of adverse effects and any other drug-related problem” (World Health Organization [WHO], 2017). Pharmacovigilance includes the retrospective collection of information from the patients’ medical records to find clues concerning a drug’s temporal association with the occurrence of adverse drug reactions (ADRs). This means finding relevant information in patient records that may support the ADR occurrence. Therefore, the development of graphical interfaces connected to CDWs can help to develop efficient and effective tools for pharmacovigilance activities. This paper presents two data visualization applications developed specifically for pharmacovigilance. The first one allows the chronological visualization of all data of a single patient in the form of a timeline; the second one is a visualization and search tool for patient sequences, called “Sequence Analyzer.”

### State of the Art

Several timeline tools for the visualization of electronic health record (EHR) data to analyze individual clinical data [e.g., Lifelines (Plaisant et al., 1998), OutFlow (Gotz et al., 2014), VisuExplore (Rind et al., 2010)] have been described in the literature. For instance, CareCruiser (Gschwandtner et al., 2011) provides the simultaneous view of the patient’s data and therapeutic protocols for the analysis of the response to treatments (e.g., for a patient on oxygen, the O_2_ saturation values will vary depending on the treatment). VisuExplore (Rind et al., 2010) offers different types of graphs (distribution curves, histograms), depending on the data type (e.g., for a diabetic patient, insulin administration is displayed as dots, while biological parameters, such as glucose concentration, are displayed as distribution curves). KNAVE-II (Shahar et al., 2006) is an interactive and semantic viewer of clinical data based on the use of domain ontologies. However, none of these tools is specific for pharmacovigilance.

Many visualization and data-mining tools have been developed for sequence analysis. EventFlow (Monroe et al., 2013) allows users to search for exact temporal patterns in a cohort and to summarize the information in the sequences. PeerFinder (Du et al., 2017) is a visual interface for finding similar individuals based on their temporal sequences. OutFlow (Gotz and Wongsuphasawat, 2012) is a tool to summarize and visualize the care trajectories of a large number of patients in the form of advanced Sankey diagrams. However, these tools were not developed for the specific needs of pharmacovigilance, and they lack functions for the search of approximate sequences and data processing modules.

## Materials and Methods

### Data Source

The data used to implement and evaluate our tools came from eHOP, the CDW technology we developed at Rennes academic hospital (France) (Cuggia et al., 2011). Briefly, eHOP integrates all types of documents produced by the hospital information system and connected with healthcare.

The eHOP CDW currently allows searching among 40 million unstructured data (e.g., clinical narrative notes, surgical protocols, X-ray, or pathology reports) and 300 million structured elements (e.g., ICD-10 diagnoses, Anatomical Therapeutic Chemical terminology for drug prescriptions and administration) from EHRs, and cover more than 1.2 million patients. Some unstructured data, such as laboratory results or diagnoses, are also recorded in a structured form thanks to the corresponding terminological codes.

### Sequence Analyzer

To analyze sequences and develop our interfaces, we defined a model of patient sequences. Each sequence includes a chronological series of events (i.e., laboratory results, diagnoses, medical procedures, the administered dose of a medication). Each event is defined by a character type, a code, a thesaurus, and a time stamp. The sequence analysis and results will depend on its content.

To analyze patient sequences, we used several algorithms. First, we implemented a modified version of the Smith-Waterman (SW) (Smith and Waterman, 1981) algorithm because it allows approximate string matching (also known as fuzzy string searching) of the reference sequences (query) with all patient sequences. We introduced modifications in the SW algorithm to take into account the temporal dimension of the data under study. We also implemented the Apriori (Srikant and Agrawal, 2018) and GSP (Srikant and Agrawal, 1996) algorithms to search for associations and common sequence patterns in the patients’ cohort.

### Timeline

**FIGURE 1:**
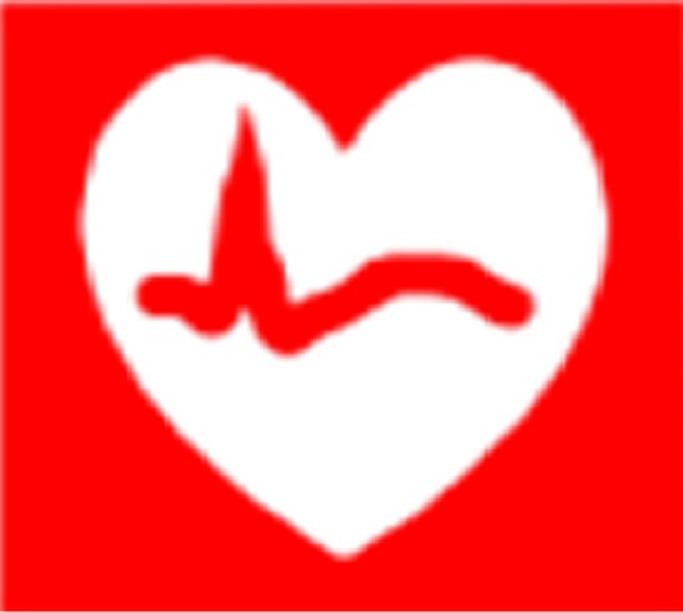
Example of VCM icon to describe a cardiac rhythm disorder.

We designed the timeline based on simple graphic semiology rules. The visual variables and data types that highlight qualitative or quantitative differences were based on Bertin’s work (Jacques, 2018). This work proposes rules for data visualization according to visual variables (forms, colors, surface, etc.). These rules allow highlighting the data qualitative and quantitative differences. The development of this timeline was inspired by the timeline proposed by C. Plaisant’s group in the Inspired EHRs ebook. We used the VCM language (Inspired EHRs, 2018) to efficiently represent the data elements in the timeline. This graphical language, which was developed by J. B. Lamy, represents the main medical concepts (symptoms, treatments, antecedents of diseases) with icons and also proposes their alignment with ICD-10 classification codes (**Figure 1**). Here, we used this alignment function to display the ICD-10 data from the PMSI database (Programme de médicalisation des systèmes d’information; medicalized information system program) in the timeline.

We developed the two prototype graphical user interfaces (GUIs) as web applications using the JavaScript d3.js (Lamy et al., 2010) and JQuery libraries for the graphical and interactive aspects. We designed the prototype components as a web service written in Java to be weakly coupled to the CDW data source (eHOP for the prototypes).

### Evaluations

#### Timeline Evaluation

Usability is defined by the International Organization for Standardization (ISO) as the “extent to which a product can be used by specified users to achieve specified goals with effectiveness, efficiency and satisfaction in a specified context of use” (Bostock, 2017). We chose the ‘think-aloud protocol’ approach that was described by Nielsen as “the most reliable evaluation method to obtain a usability estimate” (ISO, 2017). For this evaluation we recruited six users (physicians and pharmacists; mean age: 31.3 ± 4.5 years, range: 25–40) from the Pharmacovigilance Center in Rennes. Based on literature data, we determined that six people were sufficient to identify most of the timeline usability problems (Nielsen, 1993). All routinely used computers for their pharmacovigilance activity, particularly to study patient records.

To test all the timeline’s features in a real-life situation, we chose a use case in collaboration with the Pharmacovigilance Center head. Specifically, we asked the six users to determine whether the adverse reaction experienced by a patient admitted in intensive care was caused by exposure to an antibiotic. To this aim, they had to perform four main tasks: (i) to find the patient’s name and address, (ii) to determine the exact nature of the adverse reaction and collect all pertinent information, (iii) to find information on the suspected drug (name and dose, for instance), and (iv) to collect information on the ADR outcome.

After completing the tasks, each participant had to fill in the System and Usability Scale (SUS) questionnaire, translated into French. This questionnaire allows measuring the application’s usability in a fast and reliable way (Brooke, 1996).

#### Sequence Analyzer Evaluation

The aim was to evaluate the relevance of our interface for the identification of sequences that may correspond to cases of inadequate treatment. For this, we selected hospital stays from eHOP that included both international normalized ratio (INR, used to determine the effects of oral anticoagulants) measurements and vitamin K antagonist (VKA) administrations (almost 10,000 stays). The aim was to find cases of inappropriate VKA administration. VKA dosage should be reduced when INR is higher than the target value (>3). Therefore, we used as query the reference sequence “INR too high – INR too high – VKA dose increase” to retrieve patient sequences. Then, two other expert users (from Rennes Pharmacovigilance Center, but different from those used for the timeline evaluation) independently reviewed the retrieved patient sequences to classify them as corresponding, or not, to a case of inadequate treatment. The retrieved sequences were displayed first randomly and then as a sorted list according to their similarity score relative to the reference sequence.

The two users visualized and analyzed the sequences one by one, and then reported in a file the sequence identification number and whether it corresponded to a case of inadequate treatment. We timed the sequence analysis for each user and for each method (first random and then sorted list) and tested the between-method time differences with the Wilcoxon paired test. We calculated that for a statistical power of 90% with a type I error of 5% we needed a sample of 80 sequences that we randomly sampled among the retrieved hospital stays based on the similarity score against the reference sequence (query) to select sequences that were equivalently distributed in four similarity score classes ([100;75], [75;50], [50;25], [25;0]).

## Results

### Timeline Description

**FIGURE 2:**
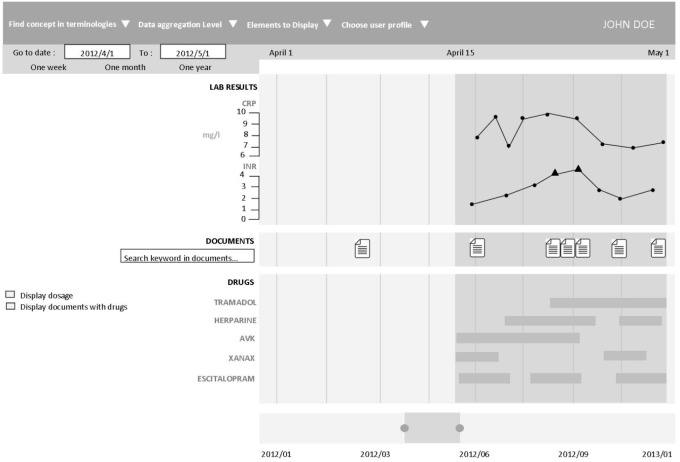
Timeline interface. The interface individual components are: (1) Selection of patient, laboratory, and clinical data and medical codes (e.g., ICD-10 code). (2) Time scale. (3) Laboratory results in the form of graphic curves (dots show normal values; triangles and their orientation represent anomalous values). (4) Drug prescriptions and treatment duration (start, end and overall duration time, as well as the drug regimen. (5) Timeline overview for navigation. (6) Overall time period selection.

The application provides access to heterogeneous clinical data information, including the form of free text (hospitalization reports, discharge letters, etc.), coded using different terminologies (e.g., the International Classification of Diseases, 10th revision, ICD-10, medical diagnosis codes), and structured data (laboratory results, prescriptions and drug administrations). Each data type has a corresponding visual representation. The prototype used a web interface to allow interactive navigation. Real patient data were used to design and evaluate for the prototype assessment. **Figure 2** is a screen mock-up to show how the information is displayed in the timeline.

To the best of our knowledge, this application includes new features. First of them, the possibility of aggregating structured data. Indeed, in the different medical terminologies, the information may appear clinically redundant. For example, at the lowest level of medication classification, Paracetamol and Doliprane© have two different codes and will therefore be on two different lines on the timeline (generic and drug princeps). However, it is the same molecule. The user has the possibility to aggregate these two data in order to display it on a single line. This semantic data aggregation/expansion was implemented by leveraging the hierarchical properties of different terminologies (Anatomical Therapeutic Chemical, ATC, classification system; French Common Classification of Medical Procedures, CCAM; ICD-10; and the local terminology used by the Clinical Laboratory of Rennes).

This aggregation function is possible for prescriptions, diagnostics and medical procedures. It allows users to interactively control the granularity of the information on the screen.

**FIGURE 3:**
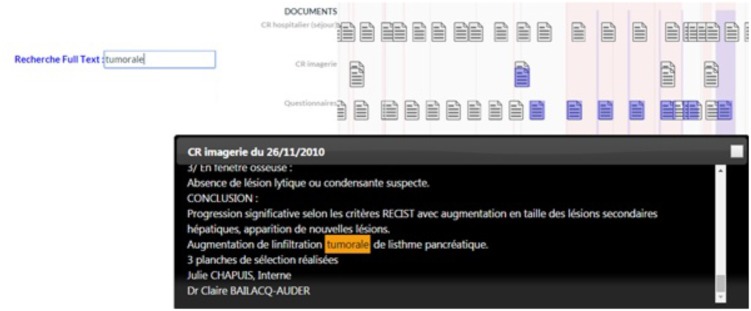
Timeline interface: Illustration of search Text Feature.

Another new feature is the display and search in free text. Free text clinical reports contain a very important source of information for pharmacovigilance activities. We have developed document search functions. A free text search field allows user to search for terms in documents. If a document contains the search term, its icon on the timeline will be colored blue. If the user clicks on the complete document, the term in the search field will be highlighted in the text as shown in the image below (**Figure 3**). The content of a document can be visible on a words cloud.

In order to help pharmacovigilants identify the treatments mentioned in textual records, we have developed NLP module which automatically extract the drug names from textual documents. This is particularly useful to find out what treatments the patient received before his or her stay in hospital, but also to find out about his or her discharge treatment.

We have also developed the ability to access, directly from the timeline, a module for visualizing the drug monography. This makes it easier to retrieve information such as half-life time or interactions with other drugs to establish the drug’s imputability for a given side effect.

All these new features were designed to facilitate navigating through the complexity of patient data.

### Timeline Evaluation

The participants’ average SUS score was 82.5 out of 100 points. After responding to the questionnaire, all participants expressed the desire to use the timeline in their daily practice. All participants could successfully perform the tasks required for the use case.

#### Sequence Analyzer Description

We developed an application to visualize sequences in a patient cohort. Unlike other visualization tools, such as PeerFinder and OutFlow, our tool manages the entire process of data visualization, from CWD data processing to screen display. The web interface is divided into two tabs: one for accessing the sequence extraction interface and the one for viewing the sequences.

**FIGURE 4:**
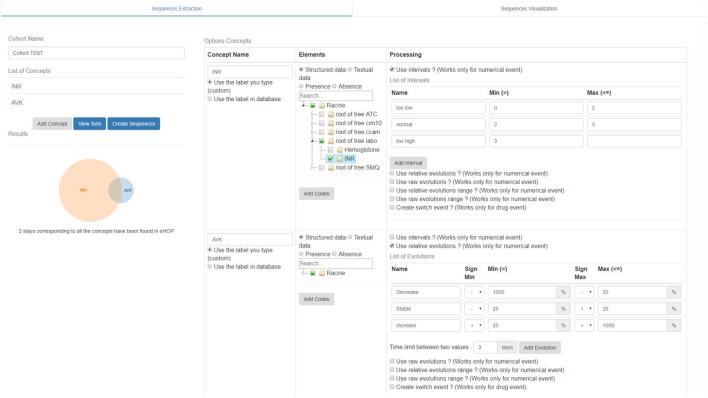
Sequence Analyzer interface: generation of sequence form.

The sequence extraction interface is used to build a cohort of patient sequences and to choose the methods for data processing (**Figure 4**). To build a cohort, the user first creates a list of concepts by choosing the codes relating to a concept in a tree structure that gathers several medical terminologies ([1] on **Figure 4**). Therefore, a laboratory code and a diagnosis code can be part of the same concept. Once the list of concepts is established (INR and VKA in the example in **Figure 4**), the user clicks on the “view sets” button ([2] on **Figure 4**). This action launches a search among the sets of hospital stays that include the concepts. The result of this search is displayed as a Venn diagram with the size of each set ([3] on **Figure 4**). This visualization mode is useful for checking whether one or more concepts are not too discriminating compared with the others. If the user is satisfied with the size of all sets, he can choose the operations for processing the data. In the example in **Figure 4**, these processing operations concern the numerical values of drug administrations and of laboratory analysis results. It is possible to discretize data from user-selected intervals, to calculate the relative or gross change of a dose or constant between events or during a time interval ([4] on **Figure 4**).

**FIGURE 5:**
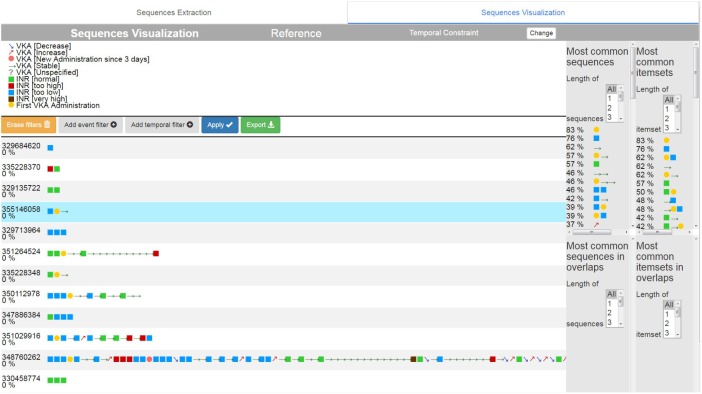
Sequence Analyzer interface: visualization of sequence.

The second tab is an interactive visual interface which was developed to visualize the sequence (**Figure 5**). A visualization dictionary has been developed for each type of event ([1] on **Figure 5**). For instance, discretized numerical events are represented by a square. Events that describe changes of a numerical variable are represented by arrows. The direction of the arrow is based on the trend of the change. Occasional events, such as a diagnosis, are represented by crosses. Each type of event has its own color. The stay sequences are represented in the main part of the interface ([2] on **Figure 5**). The results of the GSP and Apriori algorithms, which are respectively the most common sequences and associations in the cohort ([3] and [4] on **Figure 5**), are represented on the right-hand columns.

**FIGURE 6:**
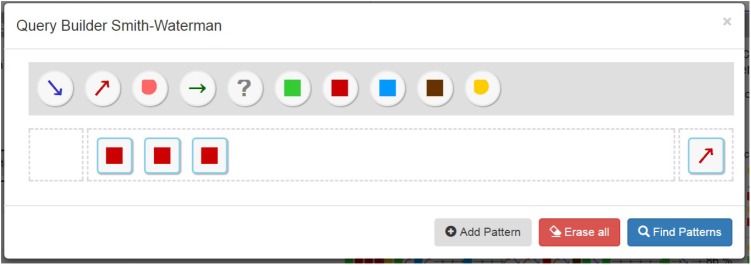
Smith Waterman Query Builder. The reference sequence includes three too high INR measurements (red squares) followed by an increased VKA dose administration (red arrow).

The user can select the reference sequence(s) to be searched in a query interface (**Figure 6**). All event types are listed in the gray area ([1] in **Figure 6**). Below this area, each line represents a search sequence ([2] on **Figure 6**). The user selects the types of events present in the gray area by a drag and drop system to constitute a sequence ([1] on **Figure 6**). The small spaces to the left and right of the search sequence are used to specify the time constraints ([3] on **Figure 6**).

**FIGURE 7:**
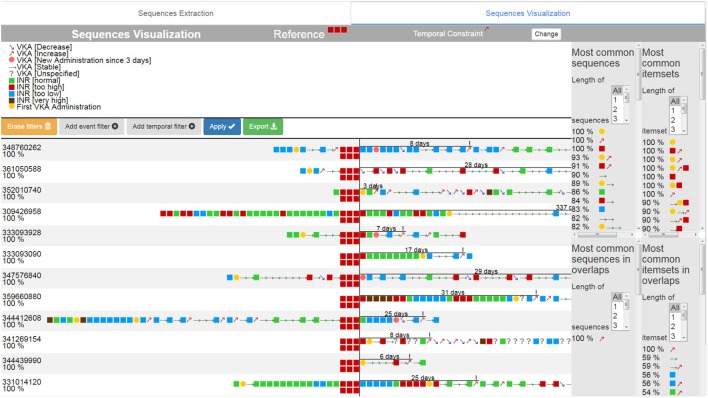
Visualization of Smith Waterman results.

The search results (**Figure 7**) are then ranked according to their similarity with the reference sequence to facilitate their visualization ([1] in **Figure 7**). The user can choose to filter the sequences according to the presence or absence of specific event types ([2] in **Figure 7**), or according to the overall duration of the sequence or the time between the aligned events and the temporally constrained event ([3] in **Figure 7**). All of these criteria can be combined.

The time interval between the alignment and the time constrained event is indicated by a line showing its duration in days ([4] on **Figure 6**). The calculation of the GSP and Apriori algorithms is restarted with only the sequences filtered, and the results are updated ([5] on **Figure 6**).

#### Sequence Analyzer Evaluation

User 1’s average analysis time of a sequence was 2.26 ± 2.86 s with ordered sequences, and 3.49 ± 3.54 s with randomly displayed sequences (*p* = 0.0003, Wilcoxon test). For user 2, the average analysis time of a sequence was 1.06 ± 1.06 s with ordered sequences, and 1.53 ± 0.85 s with randomly displayed sequences (*p* = 0.0001, Wilcoxon test).

## Discussion

### Timeline Advantages and Limits

The timeline allows to display the contents of the patient file in a chronological, structured, and concise way. Compared to the tools presented in the state of the art, the methodological contribution of the timeline are the following:

• Integration of heterogeneous data with each their corresponding visual representation (graph for biological data, VCM icons for diagnosis…);
• Data aggregation method based on medical terminologies;
• Method of searching and viewing text data;
• Pharmacology information available : NLP module which extract the drug names from textual documents and direct link to drug monography.

These functionalities have been developed specifically according to pharmacovigilance needs. The text search function makes it easy to match a medical event mentioned in a document with the evolution of the biological variable, or a drug administration. Implementing VCM logos makes it easier to identify diagnostics. Data aggregation features allow information to be synthesized to minimize cognitive load by avoiding redundant information.

Other aggregation axes could be evaluated. An aggregation between semantically and/or temporally close data could also be considered for example. This would include data that are very close in time and very similar, like repeated ICD-10 codes for chronic diseases such as diabetes. A more document oriented visualization could be proposed. Indeed, it turned out that in use the return to the text document is systematic to verify the veracity of the information.

### Timeline Evaluation

The SUS score (82.5) and the users’ feedback indicates a good usability level. This score places the Timeline in the 10% of applications with usability scores higher than 80 (Sauro and Lewis, 2012), and is higher than that of the KNAVE-II tool (69.1) (Martins et al., 2004). The success of all users in the use case tasks indicates that the timeline is suitable for linking a drug to an adverse reaction. In addition, the timeline is now used daily by Rennes Pharmacovigilance Department. However, the Timeline has not been tested with other use cases and in other pharmacovigilance services. In addition, it is difficult to quantify the time savings allowed by the timeline with this usability test. Indeed, differences in the working method of each participant have given very variable task completion times. Nevertheless, in their daily practice, practitioners appreciate the timeline for its comprehensiveness and ease of access to information.

### Sequence Analyzer Advantages and Limits

Our modified Smith Waterman algorithm has several advantages: it allows searches for similar sequential patterns. The modifications we have made allow us to take into account the time dimension of the data. Other tools (e.g., EventFlow) can search for patterns with distant events, but only exact matches. Another original feature of the tool is that it supports all the visualization steps: from searching for sequences in the database, through data processing and transformation, to on-screen display. All these steps are under user control.

However, in our case study we found that the CDW data processing step was essential for the sequence search: the sequences, and thus the patterns founded by algorithms, will vary depending on the choices made by the user. The input of experts in the field is essential to generate a sequence suitable for the research question. If the application allows searching for sequences in relation to a given pattern, there is not yet any functionality to group existing sequences together according to their similarities. Using the patterns found by the GSP algorithm as vectors, we plan to clusturize the sequences between them using k-means algorithm.

### Sequence Analyzer Evaluation

The Sequence analyzer evaluation showed that the tool allows retrieving relevant drug administration cases more quickly. This time saving is made possible by the use of the modified SW algorithm to take into account the temporal dimension of clinical data. In addition, the evaluation showed sequences that do not present the exact pattern of interest may also be relevant. For example, if we consider the first use case, the pattern “INR too high” – “INR too high” – “AVK dose administered stable” – “AVK dose administered increased,” shows an inappropriate drug administration since the administered dose is not revised downward. This is an illustration of the relevance of fuzzy research allowed by the Smith Waterman algorithm. GSP and Apriori algorithms allow patterns and associations to appear with a remarkable frequency of appearance. However, we could not test the data processing part or on another use case. The application will be tested in a broader usability test to assess more accurately the relevance of the interface before being made available to practitioners.

## Conclusion

The complexity and richness of data found in medical records require the development of tools for their efficient exploitation, particularly for viewing and querying information within a file. The Timeline and Sequence analyzer we developed are viewing and querying tools dedicated to pharmacovigilance activities. The timeline demonstrated excellent usability and was used in daily practice by pharmacovigilance center staff. The second tool allows exploring a cohort of patients by searching and visualizing their sequences. The tool has shown its interest in a performance test in the search for inappropriate prescriptions. These two tools are expected to help pharmacovigilants in their daily practice.

## Author Contributions

TL has conceived the work and ensured the development of the prototypes. TL and CP participated in the design of the prototypes. TL, CP, GB, and FT participated in the design of the studies. TL and GB performed the statistics test. TL, EP, and FT have developed the use cases. TL and MC drafted the manuscript.

## Conflict of Interest Statement

The authors declare that the research was conducted in the absence of any commercial or financial relationships that could be construed as a potential conflict of interest.
